# Examining the potential of mobile money-based health insurance for people living with HIV and hypertension or diabetes in Uganda

**DOI:** 10.3389/frhs.2026.1779532

**Published:** 2026-03-17

**Authors:** Henry Zakumumpa, Richard Ssempala, Jepchirchir Kiplagat, Japheth Kwiringira, Wilm Quentin, Verena Struckmann

**Affiliations:** 1 School of Public Health, Makerere University, Kampala, Uganda; 2 Directorate of Research and Graduate Training, Makerere University, Kampala, Uganda; 3 School of Economics, Makerere University, Kampala, Uganda; 4 College of Health Sciences, Moi University, Eldoret, Kenya; 5 Faculty of Social Sciences, Kyambogo University, Kampala, Uganda; 6 Chair of Planetary & Public Health, University of Bayreuth, Bayreuth, Germany; 7 Department of Health Care Management, Technische Universität Berlin, Berlin, Germany

**Keywords:** universal health coverage, digital technologies, health insurance, health financing, mobile money

## Abstract

**Background:**

Digital technologies are increasingly promoted as alternative pathways for financing universal health coverage (UHC) in sub-Saharan Africa, yet evidence on their acceptability among informal-sector populations remains limited. This study explored the acceptability of mobile money–based private health insurance among people living with HIV (PWH) with comorbid hypertension or diabetes in Uganda.

**Methods:**

We conducted an exploratory qualitative study in Fort Portal City, mid-western Uganda. Data were collected through four focus group discussions with PWH (*n* = 48) and 18 key informant interviews with representatives of telecom companies, private health insurers, regulators, and health providers. Data were analyzed thematically using an established analytical framework on facilitators and barriers to mobile health technologies.

**Results:**

PWH reported rising out-of-pocket expenditures for managing hypertension and diabetes compared to HIV care, which remains largely donor-funded. Facilitators to uptake included high mobile phone ownership, widespread use of mobile money, perceived affordability of monthly premiums (USD 1.35–8.20), prior experience with mobile money insurance, and convenience of digital payments. Barriers included limited understanding of insurance principles, mistrust of private insurers, fears of mobile money fraud, high internet data costs, intermittent electricity supply, and widespread poverty.

**Conclusion:**

Mobile money–based health insurance was perceived as affordable and acceptable among PWH with NCD comorbidities. However, low insurance literacy and mistrust of insurers remain major obstacles. Mobile money–based health insurance warrants further research as a complementary pathway for expanding health insurance coverage in Uganda and similar settings.

## 1 Introduction

Globally, there is growing consensus around the need to accelerate progress towards attaining universal health coverage (UHC) (1, 2). UHC has been enshrined in the sustainable development goals (SDGs). Indeed, there is increasing momentum in the global health and development agendas around the need for enhancing financial protection in accessing health care (3).

Expanding health insurance coverage is recognized as one of the important pathways for the attainment of UHC (4). There are various forms of health insurance which include employer-provided health insurance, community-based health insurance and national health insurance schemes (5).

Several countries in sub-Saharan Africa (SSA) have launched national health insurance schemes. These include Ghana, Rwanda, Kenya and Tanzania (6, 7). In Ghana, one of the countries in SSA which has a national health insurance scheme (NHIS), annual renewal of membership in NHIS can be done through digital financing platforms such as phone-based mobile money.

Digital technologies for health financing (DTHF) are becoming increasingly important in SSA. They offer significant potential for contributing solutions towards expanding health insurance coverage thereby improving access to healthcare (8, 9). DTHF has been defined broadly as any digital technology that is used to strengthen health financing systems across their three key functions, (1) raising revenues, (2) pooling resources, and (3) purchasing health services (9). In SSA, mobile money-based payment systems are surpassing payments through traditional commercial banking routes both in usage and reach (10). Although digital financing is gaining traction in SSA, there is little empirical attention exploring the potential role of digital technologies in bridging the financing gap in the quest to achieve universal health coverage particularly in the context of SSA where moves towards attaining UHC are gaining momentum (11). The importance of devising alternative financing mechanisms for achieving UHC in SSA has been heightened by the recent cuts in development assistance for health (12). In addition, there are mounting calls for pathways for bridging the financing gap for responding to the rising non-communicable diseases (NCDs) epidemic in LMICS particularly for the informal sector which has lower uptake of private health insurance (2). Studies now show that PWH who are 50 years or older are increasingly presenting with co-morbidities such as hypertension and diabetes yet there is little available financial protection for managing these multiple conditions which is a focus area for this study.

### 1.1 Uganda health financing context

Uganda's total budgetary allocation to health has been increasing over the years but spending per capita on health is steadily declining and currently stands at 7% of overall national budget commitments annually (13). The rapid growth in population at 3% per annum has resulted in gaps in health service coverage (14). Out-of-pocket payments account for one the highest sources of health financing (48%) in Uganda (14). In total, 96% of private health services are paid for by households. A 2021 report reveals that Uganda relies heavily on donor funding with National Health Accounts reflecting up to 41% of health budget funded with donor support of which 30% was off-budget support (15). Other sources of health financing include government contribution at 16% while the private sector contributed 2% of the annual health expenditure through contributions to employee health insurance coverage. With regard to the national HIV response alone, the level of donor aid has been estimated to be as high as 85% (16, 17). Since June 2004, HIV treatment has been widely available largely free of charge at public facilities across Uganda owing largely to the U.S. Presidents’ Emergency Plan for AIDS Relief (PEPFAR) (17). Before the U.S. funding freeze was announced in January 2025, PEPFAR funding provided for free-to-user HIV-specific laboratory tests such as for viral load, contribution towards procurements of antiretrovirals for 1.4 million Ugandans, training of health workers in quality HIV services delivery (18). However, there has not been similar funding priority from external donors and the national budgetary allocation with respect to the response to the escalating epidemic of non-communicable diseases (NCDs) such as hypertension and Type 2 Diabetes Mellitus (T2DM) (19). In Uganda, financial protection for managing NCDs is woefully inadequate relative to infectious diseases such as HIV, malaria and tuberculosis (19).

Against the backdrop of a large uninsured population in Uganda, health insurance entrepreneurs have devised products aimed at tapping into this large virgin market in the country. This, they have done whilst utilizing the platform provided by the two dominant private telecom companies in Uganda of South Africa-based MTN Uganda and India-based Airtel Uganda both of which have a combined a subscriber base of over 30 million Ugandans (20). Most existing entry-level mobile money-based private health insurance products in Uganda are not designed for chronic outpatient management of NCDs and mostly offer cover for emergency hospitalization. However, subscribers willing to pay much higher premiums can be covered much more comprehensively.

Although mobile money- based health insurance in Uganda holds immense promise as an alternative health financing pathway, there is little empirical attention devoted to understanding this phenomenon as it relates to expanding health insurance coverage in the Ugandan context. Research into DTHF is important to health policy and planning efforts in SSA such as by sector ministries of health and finance, global health initiatives, health insurance companies and civil society organizations engaged in advocacy for expanding access to health care. Evidence is needed to determine the affordability of private insurance through digital financing technologies, the preferences of sub-groups such as the informal sector due to the so called ‘digital divide’ (21), enablers and hindrances of the underpinning technological and physical infrastructure in SSA and understanding the commercial actors active in the health insurance space in this part of the world.

Despite the growing interest and increasingly favourable digital environment in Uganda, significant knowledge gaps remain in understanding how DTHF can be effectively and sustainably implemented and scaled-up in the context of the proposed national health scheme (NHIS) system of Uganda.

In Uganda, the national parliament approved the National Health Insurance Scheme (NHIS) in March 2019 to be financed through regular monetary deductions for employed Ugandans (6). Presidential assent to the national health insurance bill was deferred to allow for members of the informal sector, who make up the majority of the Ugandan population, to participate in financial contributions in addition to salaried Ugandans (12). Only about 2% of Ugandans have formal health insurance from private companies in Uganda (12). The majority of Ugandans who are insured are those who have formal employment. Hence most Ugandans are not insured owing to the delay in implementing a national health insurance (12).

Over the past decade, digital financing platforms have increased in popularity in Uganda (22, 23). Mobile money payments in particular are experiencing a surge in uptake. Indeed, more Ugandans make financial transactions using the mobile money platform compared to traditional banking services (22). The central Bank of Uganda is encouraging digital payments in light of the high cost of printing paper notes (23). Although Ugandans utilize mobile money for routine transactions such as paying for utility bills, paying school fees or transferring money to their relations, mobile money has not yet been adequately assessed as a platform for expanding health insurance coverage. In Uganda there has been substantial research on the subject of community based health insurance (24, 25), and a smattering of evidence on the subject of the proposed national health insurance (26). However, there is a dearth of data on digital technologies as a health financing pathway to expanding health insurance coverage in Uganda. Furthermore, there is a paucity of data exploring how digital financing technologies may potentially aid in expanding enrollments to Uganda's proposed national health insurance scheme.

We sought to explore the perspectives of people with HIV (PWH) on the acceptability of mobile money-based health insurance in terms of potential facilitators and barriers to uptake in Uganda.

## 2 Materials and methods

### 2.1 Research design

We adopted a qualitative exploratory research design (27). We sought to understand the perspectives of PWH on the potential of uptake of a mobile money based health insurance for covering health care for their comorbid conditions. Our qualitative study involved primary data collection through focus groups with PWH living with hypertension or T2DM or both. In addition, we conducted Key Informant interviews with a range of participants such as representatives of health insurance providers. This study is part of a larger project that uses a transdisciplinary research approach to explore the potential of mobile phone-based interventions to improve insurance coverage in major African cities (28).

### 2.2 Analytical framework

We drew upon an analytical framework for understanding facilitators and barriers to uptake of mobile health technologies proposed by Opoku and colleagues (29). This framework proposes three overarching themes focusing on ‘the influence of context factors’ namely a) ‘Predisposing characteristics’ of end-users of mobile technologies such as level of education and age b) ‘Need’ cluster entails influencing factors such as extreme poverty, level of awareness or information availability among end users c) ‘Enabling resources’ which has to do with the enabling physical infrastructure for mobile technologies such as electricity supply and internet connectivity. We utilized this framework in two ways. Firstly, the framework helped in the development of our Key Informant interview guide which was sectioned according to the three broad PNE themes (Predisposing characteristics, Need and Enabling resources). Secondly, we broadly drew upon the framework by Opoku and colleagues (29) in our overall synthesis and interpretation of our inductively-derived findings which reflections we present in the discussion section.

### 2.3 Study sites and study population

The study was conducted at six HIV clinics in Fort Portal City in mid-western Uganda (Table 1). We conducted the study at a regional referral hospital, three general hospitals and three sub-district health facilities (Health centre IVs). We selected Fort Port City which has an HIV prevalence rate of 18% which is three times the national average in Uganda (30). This sub-region of Uganda was purposively selected because the informal sector is dominant and also because private telecom penetration is high with two of Uganda's dominant private telecom active in this region. Furthermore, Fort Portal City is fairly representative in its peri-urban character which is similar to the majority of the urban towns in Uganda.

**Table 1:** Characteristics of participating facilities.

Health facility name	Ownership-type	Level of care in Ugandan health system	Active PWH on art (As at July 2024)
1. Fort Portal Regional Referral	Public	Referral hospital	11,069
2. Kabarole Hospital	Not for profit	General hospital	2,810
3. Virika Hospital	Not for profit	General hospital	3,414
4. Kataraka HC	Public	Health centre IV	598
5. Bukuku HC	Public	Health centre IV	1,431
6. Kaswa HC	Public	Health centre III	634

We purposively sampled PWH because studies have shown a pronounced increase in the prevalence of hypertension and diabetes particularly in those 50 years or older (31). Furthermore, new HIV therapies have been linked to particular co-morbidities such as T2DM (32) and hypertension (33) and its associated risk factors such as weight gain (34). Hence, PWH offered us a conducive comparative lens of conditions with relatively high financial protection in the Ugandan context (such as HIV treatment) (18). The management of hypertension and diabetes in Uganda does not yet receive comprehensive financial protection by the national government nor has it attracted substantial foreign aid from external donors (19). This sub-group highlights the contrasts in the challenges of filling the expanding health financing gap in SSA and the increasing need of financial protection.

### 2.4 Data collection

#### 2.4.1 Focus group discussions

We conducted four focus group discussions (FGDs) with PWH living with hypertension or T2DM or both using a pre-tested focus group guide. The pre-test enabled us identify three questions in the pilot focus group guide which were not clear to PWH during the proceedings of our pre-test. The identified questions were re-worded to improve clarity to participants in our subsequent focus groups.

With regard to the four focus groups, we conducted two gender-disaggregated focus groups (one male, one female). Each focus group entailed twelve participants. The proceedings lasted between 60 and 90 min.

We combined participants from across our six study sites and enrolled them in the four focus groups based on their demographic characteristics in terms of a) gender and b) age group. The interviews were conducted in *Rutooro* the local language spoken in Fort Portal City in mid-Western Uganda. We made a deliberate effort to be inclusive in the proceedings of the focus groups by first allowing voluntary contributions from participants. We then engaged participants directly to participate if they had not contributed to the proceedings on a specific element. We then made a point to The focus groups were led by the first author who has extensive experience in qualitative health services research. The first author was assisted by two Research Assistants (RAs). The RAs recorded the proceedings of the focus group and operated the audio-recorder. The focus groups were audio-recorded with the written informed consent of study participants.

In terms of data collection procedure, we approached the in-charge of each of the six HIV clinics and informed them about the objectives of the study and our interest of enrolling PWH living with hypertension and T2DM or both to understand how they currently finance their health care needs with respect to hypertension or T2DM compared to HIV care and treatment which is largely provided free to PWH in terms of antiretrovirals, viral load laboratory investigations and periodic clinic reviews (18). We conducted face-to-face focus groups on-site at the six HIV clinics in Fort Portal City. In terms of inclusion criteria for our focus groups, we selected PWH who were 18 years or older, those who had lived with a diagnosis of either hypertension or T2DM for at least 12 months prior to data collection. We excluded PWH who were not 18 years of age or those who did not have a diagnosis of hypertension or T2DM. Data were collected between July and September 2024.

#### 2.4.2 Key informant interviews

We conducted 18 Key Informant interviews (KIIs)with a range of stakeholders using a pre-tested interview guide. The purposively selected informants were those who were information-rich with respect to digital technologies for health financing in Uganda with particular focus on mobile money-based platforms. Broadly, we interviewed officials from the two dominant private telecom companies in Uganda (MTN Uganda and Airtel Uganda) who provide the backbone infrastructure support for mobile money services, private health insurance providers which provide financing and health insurance products (such as Sanlam Insurance) and experts on digital health financing technologies in Uganda. Table 2 shows the category of key informants we interviewed. The interviews were audio-recorded with the consent of participants. On average, the interviews lasted between 45 and 60 min. The face-to-face interviews were conducted in the offices of Key Informants by the first author who was assisted by two research assistants who took notes and operated the recorder.

**Table 2:** Category of Key informants.

Category of participants	Example of participants	*N=*
Private telecoms and insurance companies	MTN Uganda, Airtel Uganda, Sanlam Insurance	06
DHFTs regulatory authorities	Uganda Communications Commission	04
Local experts on DHFTs	Health insurance researchers	02
Health care providers	In-charges HIV, diabetes, hypertension clinics	04
Ministry of Health officials	Officials responsible for health financing	02
People with HIV	PWH with hypertension or diabetes	Four focus groups (twelve PWH in each FGD)
		18 KIs and four FGDs

### 2.5 Data analysis

We followed the procedures for qualitative data analysis recommended by Miles & Huberman (35). We followed four steps in data analysis although in practice it was a largely iterative process. We conform to COREQ guidelines for reporting qualitative studies (36). The audio recordings from our focus groups and key informant interviews were transcribed verbatim into text transcripts by a professional transcriber. In the case of the focus groups, the audio recordings were translated from *Rutooro* the local language spoken in Fort Portal City into English by a professional transcriber proficient in both languages. The transcripts were subsequently uploaded into ATLAS.ti (version 8) for data management. In the first stage of analysis, two authors read the transcribed interviews multiple times as part of data familiarization (37). In the second stage, three authors coded the transcripts and applied the resulting coding scheme to the transcripts. The codes were derived deductively from the adopted analytical framework and inductively from the data. In the third stage, the inductively derived themes were categorized under the overarching deductive themes drawn from the Predisposing Factors, Needs, and Enablers (PNE) framework that has been developed by Opoku et al., 2017 to understand the contribution of mHealth interventions to improved access to care in sub-Saharan Africa (29). The fourth stage involved overall synthesis and interpretation involving all the authors. Disagreements in the assignment of codes were resolved by consensus through a team-based process.

#### 2.5.1 Reflexivity statement

During data collection, I was mindful of being a researcher, an ‘outsider’ conducting interviews with individuals living with HIV in a Ugandan setting where HIV-related stigma is high. My initial approach was shaped by an academic understanding of ‘vulnerability’ as a passive state. However, upon reflection I conceded my own personal biases. I realized the limitations of coming from a different socio-economic standing and that I needed to be mindful of the hardships and poverty-driven realities of participants’ daily lives.

To manage my emotional response to narratives of extreme poverty and widespread social exclusion, I maintained a reflexive journal. This allowed me to separate my empathy from my analytical role. I deliberately shifted my focus from viewing participants solely through the lens of their HIV status to acknowledging their agency and resilience.

## 3 Results

### 3.1 Demographic profile of PWH in our focus groups

A total of 48 PWH living with hypertension or T2DM or both participated in our study. On average, PWH had lived with hypertension or T2DM for six years. Twelve participants had both T2DM and hypertension.

In terms of gender, 28 out of 48 (58%) participants in our focus groups were female. The majority of our focus group participants or 41 out of 48 (86%) of them were from the informal sector. Most of the participants did not have formal employment and depended on small holder crop production and livestock holdings and petty business for their livelihood. The majority had been on antiretroviral therapy for more than six years (4–12 years). In terms of age, the majority or 36 out 48 PWH (76%) were above 45 years of age. Table 3 shows the demographic profile of participants in our focus groups. All 48 participants reported owning a mobile phone with 41 participants indicating they use mobile money transactions at least twice a week.

**Table 3:** The consolidated criteria for reporting qualitative research.

No	Item	Guide questions or description	Check with a tick if implemented
Domain 1: Research team and reflexivity			
Personal characteristics			
1.	Interviewer/facilitator	Which authors conducted the interview or focus group?	√
2	Credentials	What were the researcher's credentials? E.g. PhD	√
3	Occupation	What was their occupation at the time of the study?	√
4	Gender	Was the researcher male or female?	√
5	Experience and training	What experience or training did the researcher have?	√
Relationship with participants			
6	Relationship established	Was a relationship established prior to study commencement?	√
7	Participant knowledge of the interviewer	What did the participants know about the researcher? e.g., personal goals, reasons for doing the research	√
8	Interviewer characteristics	What characteristics were reported about the interviewer/facilitator? e.g., Bias, assumptions, reasons and interests in the research topic	√
Domain 2: study design			
Theoretical framework			
9	Methodological orientation and Theory	What methodological orientation was stated to underpin the study? e.g., grounded theory, discourse analysis, ethnography, phenomenology, content analysis	√
Participant selection			
10	Sampling	How were participants selected? e.g., purposive, convenience, consecutive, snowball	√
11	Method of approach	How were participants approached? e.g., face-to-face, telephone, mail, email	√
12	Sample size	How many participants were in the study?	√
13	Non-participation	How many people refused to participate or dropped out? Reasons?	√
Setting			
14	Setting of data collection	Where was the data collected? e.g., home, clinic, workplace	√
15	Presence of non-participants	Was anyone else present besides the participants and researchers?	√
16	Description of sample	What are the important characteristics of the sample? e.g., demographic data, date	√
Data collection			
17	Interview guide	Were questions, prompts, guides provided by the authors? Was it pilot tested?	√
18	Repeat interviews	Were repeat interviews carried out? If yes, how many?	√
19	Audio/visual recording	Did the research use audio or visual recording to collect the data?	√
20	Field notes	Were field notes made during and/or after the interview or focus group?	√
21	Duration	What was the duration of the interviews or focus group?	√
22	Data saturation	Was data saturation discussed?	√
23	Transcripts returned	Were transcripts returned to participants for comment and/or correction?	√
Domain 3: analysis and findings			
Data analysis			
24	Number of data coders	How many data coders coded the data?	√
25	Description of the coding tree	Did authors provide a description of the coding tree?	√
26	Derivation of themes	Were themes identified in advance or derived from the data?	√
27	Software	What software, if applicable, was used to manage the data?	
28	Participant checking	Did participants provide feedback on the findings?	√
Reporting			
29	Quotations presented	Were participant quotations presented to illustrate the themes/findings? Was each quotation identified? e.g., participant number	√
30	Data and findings consistent	Was there consistency between the data presented and the findings?	√
31	Clarity of major themes	Were major themes clearly presented in the findings?	√
32	Clarity of minor themes	Is there a description of diverse cases or discussion of minor themes?	√

With respect to our Key Informants, the average work experience was 6 years.

### 3.2 Financial needs for controlling hypertension and diabetes

Our focus groups with PWH revealed that their out-of-pocket expenditure on health had markedly increased in the process of seeking care in the management of hypertension and T2DM. Many of the participants were on first line medication for T2DM which involved taking daily oral pills containing metformin medication. There was agreement among PWH that oral medication for diabetes was frequently out of stock across participating sites the majority of which are public facilities. For PWH living with hypertension, stock outs of the first line anti-hypertensive medication containing telmisartan and hydrochlorothiazide were said to be frequent. This, they said compelled many of them to dip into their pocket and buy from nearby private pharmacies which sell both the higher-priced brand drugs and the more affordable generic versions most of which are imported from India.

‘Whenever I go to the hospital pharmacy for Telvas H (telmisartan and hydrochlorothiazide) they tell me it is out of stock. Last week they only gave me pills lasting only one week. Stock outs are a common song at my facility. I have to get a boda boda (motor cycle taxi) to go and buy from (private pharmacies) in town’ [FGD_Male, 34, FPRH,11].

Besides medication, PWH indicated that they are required to undergo periodic laboratory tests to assess how well each condition was being managed. The most frequently mentioned laboratory investigations for those with T2DM were blood sugar tests required every three months known as the HbA1c test ($30 per test) as well as regular fasting blood sugar tests multiple times every week which costs Uganda shillings 5,000 ($1.35) per test. In addition, PWH with comorbidities are required to do regular tests to assess functioning of vital organs such as kidney or liver function. PWH with hypertension mentioned that they are required to do tests for cholesterol ($28) as well as regular blood pressure monitoring. PWH indicated that the combined costs for managing their comorbid illnesses were impoverishing them and their households. It is important to note that the majority of PWH in our study were from the informal sector with narrow resource margins.

‘’Having (high blood) pressure and sugar (diabetes) can make you poor. For diabetes I had to buy a glucometer and test strips, then I have to do the HbA1c test, then I have to order for special foods that don’t increase my blood sugar. Then I have to swallow two pills of metformin a day. Then for the (blood) pressure I have to do tests for cholesterol. I recently bought a blood pressure machine. On top of that I have to do regular tests of heart function like ECG (electrocardiogram) test. How am I supposed to afford all these things from my meagre earnings?’ [FGD_Male, 48, FPRH,13].

In a handful of PWH with poorly controlled diabetes, they revealed that complications of T2DM had resulted such as end stage kidney disease requiring thrice-weekly dialysis sessions each costing 250,000 Uganda shillings ($ 68) per session which is equivalent to an average monthly salary in Uganda. There were four participants who reported partial eye sight loss known as diabetic retinopathy, a complication of diabetes, which required surgery costing at least 21 million Uganda shillings ($ 5,675) at one provider in the Ugandan capital. This they said they couldn’t afford and as such had postponed the procedure. The costs of seeking expert care from consultant cardiologists or diabetologists were said to be higher than those for seeing a general practitioner (GP). Because many PWH were unable to afford the costs of managing their co-morbid illnesses they were compelled to seek herbal remedies that are common in Ugandan traditional cultures. Against this backdrop of gaps in financial protection from out of pocket expenditure for managing their comorbid illnesses, we explored the perspectives of PWH regarding the potential uptake of mobile money based health insurance.

### 3.3 Facilitators

Table 4 below shows that there were multiple perceived facilitators of the uptake of DTFHs.

**Table 4:** Facilitators and barriers to DTHF uptake categorized under the opoku framework.

PNE framework theme	Facilitators	Barriers
Predisposing factors	High use of mobile money payments	Insurance principles
	Affordability of monthly payments	Widespread extreme poverty
	Prior insurance experience	
Needs	Convenience of payment mode	Mistrust of insurance providers
Enabling resources	Wide digital footprint	Data security
		High cost of internet data
		Intermittent electricity supply

#### 3.3.1 High use of mobile money payments

Participants indicated that mobile money use in daily financial transactions was high as evidenced in their printable mobile money statements which they consented for us to review. We found that all participants in our focus groups had mobile money accounts and they all utilized mobile money payment systems on an almost daily basis. In Uganda, having a mobile phone connection or sim card from a telecom provider automatically enables you to have an individual mobile money wallet.

‘Mobile money is part of my everyday life. Mobile money is my bank account. I pay for almost everything using momo (mobile money). I don’t keep too much cash on me anymore. When I go to the shop at the corner to buy bread and sugar I pay using momo. When I need to send money to my daughter in boarding school I send the money to her class teacher’s number. I can’t imagine a life without momo anymore’ [FGD_Male, 48, FPRRH,04]

Participants identified the high mobile money account coverage for the over six million Ugandans with a phone connection makes the option of monthly monetary deductions for health insurance possible.

Key Informant interviews with officials from private telecoms concurred with the notion that high phone ownership rates and widespread use of mobile money payments systems made it a conducive platform for phone-based health insurance.

‘Uganda has high phone ownership and it is even going up. More Ugandans own a mobile money account than those who own a conventional bank account. Mobile money is part and parcel of Ugandan life. People make deposits and withdrawals on mobile money. We find that this an excellent platform to tap into for expanding private health insurance especially for the informal sector in Uganda’ [KII_Male,49, Telecom official, 03]

#### 3.3.2 Affordability of monthly payments

Although there is widespread income poverty across Uganda, there was a sense among PWH that monthly deductions from their mobile money accounts for purposes of paying for health insurance for managing their co-morbidities could be afforded from their disposable income which is a revelatory finding of this study. Overall, PWH indicated that they were able to afford monthly mobile money deductions of 5,000 Uganda shillings ($1.35) for health insurance coverage.

‘I think I can afford to have monthly deductions made from my mobile money account to pay for health insurance of about 5,000 shillings ($ 1.35). I am comfortable with that sum. I would be okay with it being removed from my mobile money account every month.’ [Female, 47, FPRRH,07]

However, a section of participants indicated being able to afford a higher monthly premium which is perhaps unsurprising given the variations in individual incomes among our participants.

‘For me I can afford to set aside at least 30,000 Uganda shillings ($ 8.20) every month to pay for health insurance. Yes, I can instruct the insurance company to deduct that amount from my mobile money account every month. I think I am comfortable with than amount. That one I can sustain every month’ [FGD_Female, 38, FPRRH,09]

Overall, PWH were willing to pay a monthly contribution towards their health insurance from a private health insurance provider through deductions from their mobile money account.

Key Informant interviews with officials from telecom companies and private insurance companies revealed that the indicated monthly deduction of 5,000 Uganda shillings ($1.35) was comparable to the fees they charged for mobile money based health insurance. For instance, ‘Dwalilo care’ provides for a minimum of $3 dollars momo deduction per month (38). For AYO, the minimum contribution to their flagship health insurance product is Uganda shillings 120,000 ($33) (38, 39). The contributions are made to this wallet each time subscribers buy airtime for phone calls from their mobile money accounts (40).

#### 3.3.3 Previous experience with mobile money based health insurance

During the proceedings of our focus groups it emerged that a handful of PWH had prior experience of being enrolled on mobile money based health insurance provided by private telecom companies. The most cited mobile money based health insurance was AYO an offering from MTN Uganda.

Two PWH recounted experiences of receiving cash payments for their health insurance claims from their AYO mobile money based medical cover as promised in the medical emergencies they encountered. One of the medical emergencies involved hospitalization after a severe illness and another involved a serious road traffic accident which involved hospitalization as recounted by one of the participants below:

‘Last year I was involved in a nasty accident in a kamunye (mini bus commuter taxi). I woke up to find myself in a hospital bed. I stayed in the hospital for three days. I paid a lot of money to the hospital because I suffered multiple fractures to my leg. Fortunately, I was insured at the time of the accident by AYO of MTN Uganda. I took my receipts from the hospital and AYO reimbursed me without much hassle. So, it is true that health insurance can be a savior in times of unforeseen medical emergencies’ [FGD_Male, 47, FPRRH,06]

There was a sense among some participants that their previous experience demonstrated that mobile money based health insurance was effective in cushioning them from heavy financial expenses associated with hospitalization based on their lived experience. They indicated that from personal experience, mobile money based health insurance had demonstrated to them that there were tangible returns that accrue and that they therefore had no qualms about paying for health insurance cover to help them manage their co-morbid illnesses.

#### 3.3.4 Convenience of payment mode

A notion that was frequently mentioned by PWH was that paying for health insurance via mobile money was faster, much more convenient and preferable to seeking conventional private health insurance which was perceived to involve more procedures such as filling paper work and making in-person visits to offices of insurance companies. PWH indicated that paying for their health insurance via mobile money saves them time and transport than would have otherwise been spent enrolling for the health insurance. The sheer convenience of payment via a phone when frequently mentioned as described by a PWH below:

‘‘It is incredibly simple. All I do is press a couple of buttons on my phone and I am covered for medical emergencies. I don’t have to fill a form and give mundane details such as ‘mother’s name, fathers name’. It is so easy and convenient for those of us who are pressed for time’’ [FGD_Female, 36, FPRRH,02]

PWH indicated that paying for health insurance through mobile money was easy to use. It was mentioned that the steps for enrolling for health insurance were uncomplicated for the health insurance products offered on Ugandan telecom platforms such as AYO by MTN Uganda and *Dwalilo*care on Airtel Uganda.

### 3.4 Barriers

#### 3.4.1 Limited understanding of insurance principles

It emerged in our focus groups, that PWH had little awareness of the principles of insurance. A common misconception was that refunds should be paid to those who are insured if they do not fall sick during the period they were covered by health insurance providers as illustrated in the quote below:

‘If I pay money to be insured for my health for one year and in that year I don’t fall sick then the insurance company should refund my insurance money. What value do I get if I am insured every year for three years and in all that time I don’t fall sick?’ [FGD_Female, 44, FPRRH,11]

One of the participants who had enrolled with AYO health insurance appeared oblivious to the fact that the insurance company would only cover expenses relating to hospitalization and excluded out-patient visits.

Our participants appeared oblivious to the basic principle of ‘indemnity’ or that they would only be paid the equivalent of the loss they incurred. As has been noted ‘In other words, the insured should be compensated the amount equal to the actual loss and not the amount exceeding the loss’. There was little knowledge of the basic concept of ‘risk pooling’ by a group as well as the need of insurance companies to survive in business by managing pay outs.

#### 3.4.2 Mistrust of insurance providers

Mistrust in private insurance companies in Uganda was common in PWH. There was a widely held perception that local private insurance companies in Uganda avoid paying claims. PWH perceived private insurance companies in Uganda to be driven by self-interest or profit maximization at expense of their insured clients.

Many of the PWH had misgivings about the willingness of Ugandan insurance companies to make payments if they are actually hospitalized and were deserving of insurance pay outs.

‘I don’t trust insurance companies in Uganda. They avoid paying claims. My worry is you may pay for the health insurance but they will decline to refund you even who you genuinely fall sick and were admitted to hospital. They may give excuses that that type of illness is not covered by them or that hospital is not on their approved list. They can come up with many excuses when it comes to pay’ [FGD_Female, 52, FPRRH,08]

Mistrust emerged as a major barrier to the potential uptake of private health insurance as the PWH expressed apprehension at the likelihood of Ugandan private insurance companies declining or delaying payouts even in genuine cases deserving of payment.

#### 3.4.3 Data security of mobile money payment systems

A recurring theme in our focus groups were fears of fraud associated with mobile money transactions or digital financial services in general. There was with a widely held perception that the backbone infrastructure in Uganda was not secure enough and did not instill confidence among users in mobile money payment systems. Participants identified a plethora of concerns around cyber security such as identity theft, sim card phishing or fraudulent withdrawals which were said to be common. PWH perceived authentication protocols for making mobile money payments to be weak. In Uganda, making a mobile money transaction requires only a single five-character password which is meant to be known to only the account holder however it is not uncommon to share passwords with family.

‘Improving the security of mobile money transactions is a top priority for us. We impart upon our clients the importance of not sharing their passwords and being absolutely sure about the person or entity they are sending money to. We are constantly working for ways for enhancing the security of mobile money business processes to in order to instill confidence in our clients that their transactions are secured’ [Representative of telecom companies, 04]

In addition, mobile money payments are based on providing correct details of the phone number of the payer and the payee. In the event where the payer enters an incorrect phone number of the beneficiary of a mobile money payment and the funds are successful remitted to the provided incorrect number, these monies can be reversed back to the sender if the telecom company is notified immediately.

Sending mobile payments to the wrong phone number is common in Uganda however reversals are not currently possible if the wrong recipient withdraws the money before recovery measures are instituted. These underpinning constraints around the security of mobile money payments played into fears among PWH around enrolling for health insurance using the mobile money platform.

An official from one of the leading telecoms in Uganda indicated that they were continually devising ways of improving the security of their systems and mentioned that they have now introduced a recall function by the sender if they realize they have sent money to the wrong person.

‘We are continually devising strategies to improve the security of our mobile money payments systems. We have introduced a recall function in case a client sends money to the wrong phone number. They can recall that money in seconds immediately they discover the recipient is not the intended one. We also thinking around strengthening our authentication protocols’ [[KII_Male,49, Telecom official, 04]

Issues surrounding data security of mobile money payments were prominent in the minds of PWH in our focus groups. This, points to the need for their prioritization by telecoms and regulatory authorities to enhance uptake of mobile money based health insurance in the Ugandan context.

#### 3.4.4 High cost of internet data

There was unanimity among PWH that the cost of internet data was high across internet service providers in Uganda. PWH identified this as a barrier to utilizing their phones for internet-supported applications including potentially for enrolling for mobile money based health insurance. The high cost of internet, they maintained, was why low-income earners in Uganda use their phones largely for voice calls and not for internet-supported applications or merely surfing the internet.

‘Officials from Uganda Communications Commission (UCC) which retains regulatory oversight of the digital and telecom sector in Uganda concurred and conceded that the cost of internet data was high in Uganda even when compared to her neighbors such as Kenya, Rwanda and Tanzania.

‘Lowering the price of mobile internet is one of our foremost priorities as a regulatory agency of government. We have implemented several policy interventions aimed at lowering the cost of data to promote affordability by the ordinary Uganda. We also support bringing on board more internet service providers in order to influence a price reduction. But there are other supply-side issues such as the investments by internet service provider in the latest technology such from transitioning from 4G to 5G and the need to recoup their investment’ [KII_Male,51, Telecom official, 02]

#### 3.4.5 Intermittent electricity supply

Electricity supply coverage in mid-western Uganda was highlighted as a potential draw back in efforts to promote uptake of mobile money based health insurance. PWH reported that electricity supply was only available within the city and that those who lived in the peri-urban outskirts of the city were not yet connected to the national grid. It is important to note while the majority may derive their living from the city the majority find affordable low-cost housing in the outskirts of the city. Weak electricity coverage outside of the city was raised as a major constraint to charging phones which limited their use in daily life for many potential health insurance clients. One of the participants highlighted the dire constraints of having to travel hundreds of kilometers to an urban site to be able to charge their phone to be able to operate it on a routine day.

‘When I need to charge my phone, I am forced to get a boda boda (motor cycle taxi) to take me into town which costs me 20,000 shillings ($5.46). Where I reside, the electricity is always on and off. I incur transport costs whenever I have to take my phone for charging in the nearest town. Sometimes my phone is off for two days or more because I need to charge it’ [FGD_Female, 33, FPRRH,01]

#### 3.4.6 Widespread extreme poverty

PWH frequently cited the widespread extreme poverty in mid-western Uganda as a major limiting factor in the potential uptake of mobile money based health insurance. Participants highlighted the fact that the majority of ordinary Ugandans in this part of the country are poor and belong to the informal sector with the majority engaged in subsistence agriculture or petty trade. Those who had meaningful disposable income were said to be the minority. The widespread extreme poverty was identified as a hindrance in terms of affordability of monthly contributions to a health insurance fund.

‘I think many people would like to be insured in terms of their health but our people are poor. They are grappling with very basic needs such as finding enough food to eat and paying school fees for their children. They are worried about today and not tomorrow. They are worried about where lunch will come from today and not what will happen when I fall sick in December’ [FGD_Female, 52, FPRRH,08]

The notion that ordinary Ugandans in this part of the country have meagre disposable income and that most of their earnings go into paying for basic necessities such as buying food and that there was barely enough left to contribute to paying for private health insurance was prominent in our discourse with PWH.

A related notion was the sense among participants that funding health care is a primary duty of the national government and that individual private health insurance should not be necessary. A section of PWH suggested that in the context of HIV services a dependence syndrome on external donors had coalesced among Ugandans which dampened their appetitive for dipping in their pockets to pay for their health care.

## 4 Discussion

We set out to explore the perspectives of PWH regarding the potential of uptake of mobile money based health insurance in Fort Portal City in mid-western Uganda. We identified potential facilitators which include prior experience with mobile money health insurance, high mobile phone ownership, availability of mobile money-based health insurance products, perceived affordability of a monthly monetary contribution towards health insurance and perceptions that mobile money health insurance is easy to use and more convenient than conventional private health insurance. On the other hand, we found several potential barriers which include low awareness of insurance principles, the high cost of internet data in Uganda, mistrust in private insurance actors in Uganda, fears of fraud in mobile money transactions and intermittent electricity supply.

Our findings add to filling the void in the literature on digital health financing technologies in Uganda with potential relevance to countries with similar settings (22, 41, 42). Although there is a paucity of evidence on DTHF in Uganda, previous studies in Kenya and Nigeria have identified data security in digital financing mechanisms as a teething challenge deserving of policy and programming interventions in promotion of consumer protection (43). A previous study by Kiguba and colleagues in Uganda found that physical infrastructure limitations such as unreliable electricity supply in Northern Uganda were a hindrance to uptake of digital health technologies (44). Our study adds to evidence suggesting that high mobile phone ownership is a facilitator to the scale up of digital technologies in SSA (45).

Our findings add to the mounting evidence of the growing importance of mobile money payments by the informal sector in SSA relative to formal financial services such as banking (46). Studies have highlighted the increasing adoption of mobile money payments as a preferred financial transaction platform in SSA and its importance in deepening financial inclusion particularly among the poor. Our literature review revealed that in some parts of SSA, more individuals own a mobile money account than those who own a traditional bank account. In our sample of PWH we found that nearly all of them owned a mobile phone and that they utilize mobile money transactions on an almost daily basis. A highlight of our findings is the experiences of PWH with mobile money phone health insurance for which there has been a paucity of data. In addition, our study fills a void in the literature on the phenomenon of digital health financing technologies in Uganda for which there is a dearth of empirical data (10).

### 4.1 Implications for increasing financial protection for non-communicable diseases

In this study PWH with co-morbid conditions reported high out of pocket expenditure for expenses incurred in seeking care for controlling hypertension and T2DM due to limited financial protection by the national government. An important contribution of this study is in providing human-centred profiles of the financial constraints of living with non-communicable diseases in Uganda. Our study elicits the plethora of health care needs for managing NCDs in Uganda. PWH identified stock outs and shortages of medications for hypertension and medication that required them to dip in their pockets and buy the needed medication from private pharmacies. These challenges were compounded for PWH with poorly controlled diabetes and hypertension which resulted in complications. Our study therefore engages on the subject of health insurance needs for managing NCDs particularly for informal sector members who dominate in the study area we focussed. Studies in East Africa have documented the catastrophic expenditure associated with complications with T2DM such as end stage kidney disease (47, 48). Previous studies in SSA have highlighted the individual and household level financing constraints of seeking health care for diabetes and hypertension (49, 50).

### 4.2 Policy and programming implications

Our study has implications for strengthening the legal and regulatory framework underpinning digital health financing technologies in Uganda and other countries with a similar setting. Local stakeholders including regulatory officials pointed to the need for interventions aimed at lowering the cost of internet data such as through as through tax measures which would promote financial inclusion (51). Matovu and colleagues (52) have highlighted the importance of strengthening security for mobile money payments systems to instil confidence in consumers and protect them from financial loss accruing from fraudulent transactions a topical subject in the literature (43, 53).

Our study highlights the low awareness among informal sector members around the principles of insurance in general which emerged as a potential barrier to uptake of mobile money based health insurance in Uganda. This finding points to the need for educating the masses in SSA around the insurance education as a means of enhancing uptake of health insurance which would in turn support the global health and development agenda of accelerating progress towards universal health coverage (54) Studies in SSA have identified low knowledge on basics insurance principals as a hindrance to enrolment in insurance schemes (55–57). In this study we found that PWH were distrustful of private insurance companies which they perceived as profit oriented and disinclined to paying claims.

On the whole, we find congruence with the framework by Opoku and colleagues (29) proposed for understanding enablers or hindrances to uptake of digital health technologies such as with regard to the theme of ‘predisposing characteristics’ such as age, education and social class as ‘drivers’ of uptake of digital health technologies. We found empirical support for the framework's theme of ‘need’ with respect to barriers to uptake of DTHF such as widespread extreme poverty and lack of information on basic principles of insurance among PWH. With respect to the theme of ‘enabling resources’ we found support in our study for physical infrastructure limitations such as intermittent electricity supply and the poor quality of internet connectivity in mid-western Uganda as perceived barriers to uptake of mobile money-based health insurance.

### 4.3 Strengths and limitations

Our study had several strengths. These include the use of an analytical framework in the study design and triangulated data collection such as through Key Informant Interviews and Focus Group Discussions. We provide a patient-centred, in-depth account of challenges of financing health care for NCDs in a low-income setting and the potential of ameliorating the financial hardships through relatively affordable mobile money insurance particularly for the informal sector. We attempt to fill a void in the literature on digital health financing technologies in Uganda and the potential of uptake of mobile money insurance for which there has been little research. Our qualitative study has limitations in generalizability of our study findings. Given that we focussed on one geographic sub-region of Uganda and that our participants were purposively selected our, study findings need to be interpreted in light of the sampling limitations. Secondly, the majority of facilities we went to were public facilities. Perhaps including a larger sample of private facilities would have provided a more rounded analysis.

## 5 Conclusion

Overall, PWH and officials from telecom companies perceived a monthly mobile money deduction towards health insurance as affordable and acceptable to PWH living with hypertension or diabetes. However, mistrust of insurance companies and limited awareness of the principles of insurance pose a major barrier to potential uptake. Our findings suggest that mobile money based health insurance is worthy of further research as a pathway to expanding health insurance coverage in Uganda and similar settings.

## Data Availability

The original contributions presented in the study are included in the article/Supplementary Material, further inquiries can be directed to the corresponding author.
